# Hepatic mesenchymal hamartoma: An uncommon but important paediatric diagnosis

**DOI:** 10.4102/sajr.v24i1.1891

**Published:** 2020-07-27

**Authors:** Sheree C. Gray, Jacobus A. Pienaar, Zelia Sofianos, Jacob Varghese, Ilonka Warnich

**Affiliations:** 1 Department of Radiology, Faculty of Radiology, Klerksdorp/Tshepong Hospital Complex, Klerksdorp, South Africa; 2 Department of Radiology, Faculty of Radiology, University of the Witwatersrand, Klerksdorp, South Africa; 3 Department of Radiology, Faculty of Radiology, Klerksdorp/Tshepong Hospital Complex, Johannesburg, South Africa; 4 Department of Radiology, Faculty of Radiology, University of the Witwatersrand, Johannesburg, South Africa

**Keywords:** Hepatic mesenchymal hamartoma, Paediatric population, Cystic liver masses, Hepatic tumours, Paediatric alfa fetoprotein levels, Computed tomography, Ultrasound

## Abstract

Hepatic mesenchymal hamartoma is a rare hepatic tumour seen in the paediatric population. Although this entity has a variable imaging appearance, it has a favourable prognosis if diagnosed and managed correctly. This case presents the ultrasound and computed tomography findings of an 11-month-old patient who presented with a history of progressive abdominal distension and an elevated alfa fetoprotein level on biochemistry. The case describes how a confident perioperative diagnosis could be made on the basis of characteristic imaging features.

## Introduction

Hepatic mesenchymal hamartoma is an uncommon, benign, paediatric tumour, most commonly encountered during the first 24 months of life. Because of an excellent prognosis, distinguishing this tumour type from other liver masses is essential, and confident perioperative diagnosis could be made on the basis of characteristic imaging features.

## Case presentation

An 11-month-old male patient presented at the department of paediatrics with a 3-month history of progressive abdominal distension. The child was otherwise systemically well, born at term and had no significant background medical history. Clinical examination revealed an upper abdominal mass lesion, non-tender and firm to palpation. Ensuing abdominal ultrasound showed a complex solid-cystic upper abdominal mass lesion, with concern for possible hydatid disease. Initial biochemistry revealed mild elevation in gamma-glutamyl transferase (GGT) with a marginally elevated alfa fetoprotein (AFP) level of 44.4 µg/L (reference range: 0.6 µg/L – 7.9 µg/L, as per the National Health Laboratory Service [NHLS]). Hydatid serology, blood cultures and confirmatory human immunodeficiency virus (HIV) testing were negative. Further imaging was then preformed.

**FIGURE 1:**
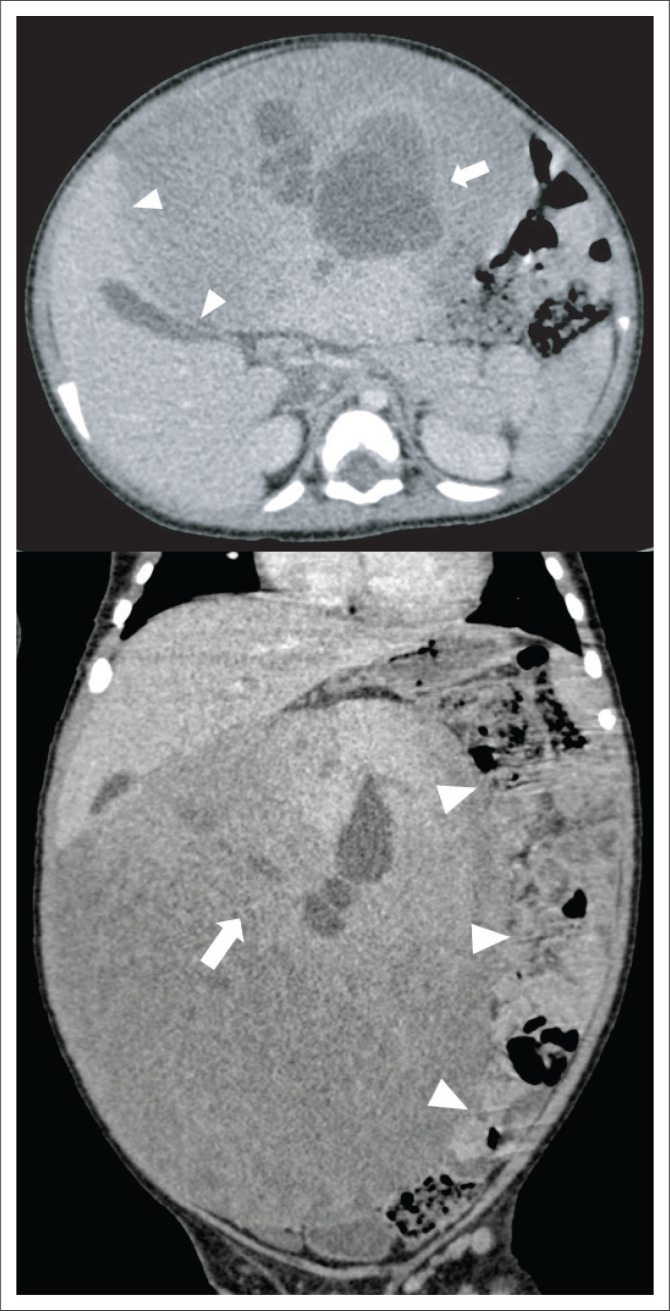
**Initial imaging –** Axial and coronal contrast-enhanced computed tomography of the abdomen (portal venous phase) demonstrates a large, complex mass lesion in the right abdomen. It abuts the inferior liver border with a poor plane of separation and displaces the bowel to the left (arrowheads). There is a lack of peripheral hepatic parenchymal extension along the edges of the mass (claw sign); therefore, hepatic origin cannot confidently be suggested. Solid and cystic elements are clearly distinguishable (arrow) with the classic ‘swiss cheese appearance’.

Contrast-enhanced abdominal computed tomography (CT) in the portal venous phase demonstrated a large, well-demarcated intraperitoneal mass (Figure 1), measuring approximately 17 cm × 16 cm × 15 cm. The mass was localised within the right abdomen, displacing the bowel to the left. It was abutting the inferior border of the liver with a poor plane of separation. However, there was no hepatic parenchymal claw sign, precluding a confident diagnosis of hepatic origin. The mass extended into the central abdomen as well as into the flank and pelvis on the right. It showed an inhomogeneous attenuation pattern with moderately enhancing soft tissue components, large low attenuating cystic areas and low attenuating tubular structures. No overt calcification or fat elements were demonstrable. The lesion significantly displaced the surrounding viscera, with a degree of bowel attenuation, but there was no bowel obstruction or features of direct local invasion. Minimal abdominopelvic free fluid was noted and no significant lymphadenopathy was visualised. The gall bladder, hepatic artery and portal venous system were normal, with other imaged structures unremarkable.

Based on the above-mentioned imaging findings, the initial differential diagnoses included that of primary hepatic lesions as well as extra-hepatic mesenteric lesions. The most likely diagnosis within each category was a hepatic mesenchymal hamartoma and mesenteric lymphatic malformation respectively.

**FIGURE 2:**
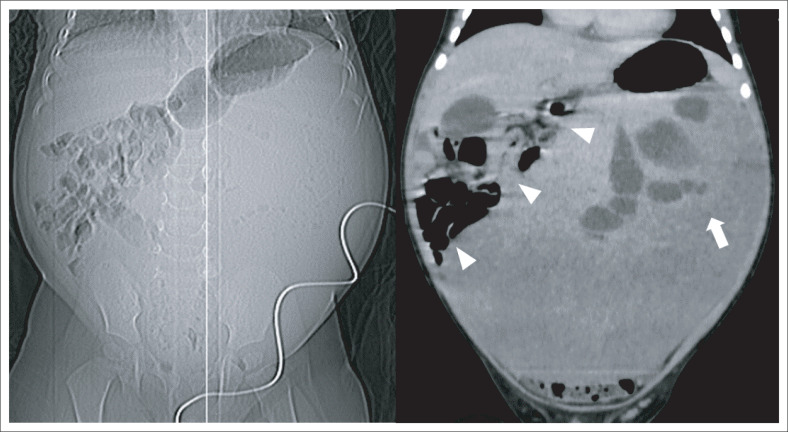
**Follow-up imaging –** Abdominal computed tomography scout image and coronal contrast-enhanced imaging (portal venous phase) demonstrates the mass to have shifted into the left abdomen (arrow), causing displacement of the bowel to the right (arrowheads). This reveals the mass to be mobile and separable from the right and left liver lobes.

**FIGURE 3:**
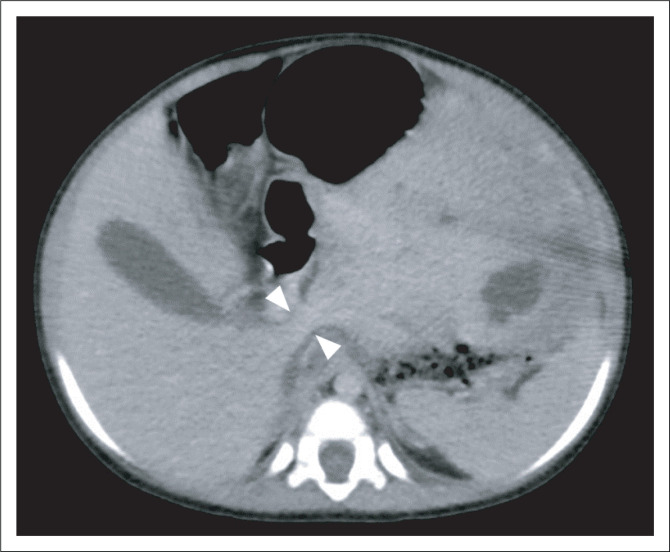
**Follow-up imaging –** Axial contrast-enhanced computed tomography of the abdomen (portal venous phase) shows the mass within the left upper abdomen. A thin pedicle is seen connecting the mass to the caudate lobe of the liver (arrowheads). This confirms a pedunculated mass of hepatic origin.

The child was referred to a tertiary institution for further management. Because of delay in follow-up, a repeat abdominal CT was performed approximately 4 weeks after the initial study. This was carried out to evaluate for interval growth and assist in surgical planning. It revealed the mass to have shifted into the left-sided abdomen, completely separable from the right and left liver lobes, with displacement of the bowel to the right (Figure 2). A thin pedicle could now be identified connecting the mass to the caudate lobe of the liver (Figure 3). The mass showed no interval change in size. A pedunculated hepatic mesenchymal hamartoma was confidently diagnosed, with no need for further imaging. Given the lack of markedly raised AFP levels, a hepatoblastoma was a less likely, however, still important in the differential diagnosis.

Subsequent laparotomy confirmed a large pedunculated mass arising from the caudate lobe of the liver. The lesion was excised and sent for histological evaluation. Macroscopic pathological assessment showed solid components with the appearance of haemorrhagic liver parenchyma with areas of haemorrhage as well as cystic components, the largest measuring 30 mm × 25 mm × 20 mm. The cysts contained yellow serous fluid with a smooth, unilocular inner cystic wall. Areas of necrosis and calcification were not identified.

Microscopic and immunohistochemical evaluation showed clusters of hepatocytes, without cellular atypia, along with numerous bile ducts of variable proportions, haphazardly arranged within an oedematous fibromyxoid stroma. The stroma consisted of spindled fibroblasts with numerous vascular spaces interspersed with the bile duct. The cysts were lined with a single layer of columnar epithelium, also without atypia. Areas of acute haemorrhage were noted, with prominent vascular ectasia. No granulomatous inflammation, viral cytopathy, parasites, protozoa or features of invasive malignancy were visualised. These findings are in keeping with a histological diagnosis of hepatic mesenchymal hamartoma.

A 3-month follow-up ultrasound revealed a normal liver with no residual or recurrent tumour.

### Ethical considerations

This article followed all ethical standards for a research without direct contact with human or animal subjects.

## Discussion

Hepatic mesenchymal hamartoma is an uncommon, benign tumour with an excellent prognosis, which may be encountered during paediatric imaging, typically in patients aged between 1 month and 4–5 years.^1^ A slight male predominance is documented. As the term ‘hamartoma’ implies, it should rather be considered a developmental malformation than a true neoplasm. Very rarely these hamartomas can undergo malignant degeneration into undifferentiated embryonal sarcoma. Mesenchymal hamartomas were first described in the literature by Edmondson in 1956.^2^ Initially, these lesions were variably named as hepatic or giant lymphangiomas, bile duct fibroadenomas, and cavernous lympangiomatoid tumours before being grouped together under the term ‘mesenchymal hamartoma’. Association with polycystic kidney disease, congenital hepatic fibrosis and biliary hamartomas has been described.^1,3,4^

Clinical presentation varies on the basis of tumour size. Smaller hamartomas are usually asymptomatic, with a painless mass lesion incidentally discovered on abdominal examination. Pathologically, the neoplasm develops and progressively enlarges because of mesenchymal proliferation, with the development of multiple fluid-containing cysts contributing to the rapid enlargement of mass. Whilst well-circumscribed, this mass is unencapsulated.^5,6^ Serum AFP levels can be within the normal limits, but can also be elevated because of the proliferation of hepatocytes in the neoplasm.^5,7^ Elevated GGT, but otherwise normal liver function tests, is also reported.^1,3^

Regarding serum AFP levels in the paediatric population, these levels are routinely used as an important biomarker for hepatoblastoma, where values are typically very elevated (up to approximately 100 000 times the normal value). However, this is limited in terms of sensitivity and specificity for the diagnosis of malignancy, as it is a tumour-associated and not a tumour-specific protein. A mild elevation in serum levels can be seen with other malignant and non-malignant conditions, such as hepatocellular carcinoma, mesenchymal hamartoma, cirrhosis, viral and chronic active hepatitis, and ulcerative colitis, to name but a few. In patients with hepatoblastoma, 5%–10% of these may have a low or even normal AFP level. Concentrations are also age-specific, with high levels observed in neonates and exceedingly high levels in preterm neonates. Levels usually decline and reach normal concentrations at 8–12 months of age. Alfa fetoprotein levels should thus be correlated with imaging and histopathology findings whilst taking the patient’s age into account.^8,9,10^

Mesenchymal hamartomas are commonly large at the time of diagnosis, weighing over 1 kg, and most commonly arise from the right lobe of the liver. These lesions arise from the mesoderm within portal tracts. Pathological hallmarks are clusters of normal hepatocytes in a matrix of porous mesenchyme, which allows accumulation of fluid as it degenerates, predisposing to cyst formation. Additional cystic elements are attributable to dilated biliary ducts.^1^

Radiological imaging plays a major role in both diagnosis and planning the surgical approach to the lesion, as clinical and biochemical features are nonspecific.^2^ Plain abdominal radiographs may show hepatomegaly or a paucity of bowel gas in the right upper quadrant, suggesting a hepatic mass lesion. Sonography is the modality of choice for initial assessment in the paediatric age group. Right upper quadrant findings can vary from large, septated cysts to small cysts with thick septations, with varying degrees of associated solid echogenic tissue, in relation to the liver and separable from the biliary tree. Computed tomography confirms sonographic findings, and better delineates the extent of the mass lesion and relationship to other intra-abdominal viscera. The appearance is commonly that of a well-defined solitary mass lesion of hepatic origin, either completely intrahepatic, partially extrahepatic, and with an exophytic or pedunculated pattern of growth. Enhancing soft tissue elements are usually seen surrounding a central area of simple fluid attenuation, with variable degrees of internal septation and loculation. This multi-cystic configuration is commonly labelled as a ‘swiss cheese appearance’. Completely solid, stromal predominant tumours are rarely seen and are usually smaller lesions. Haemorrhage is uncommon. Calcification, although it may very rarely occur in these lesions, is much more commonly seen in other tumours. Computed tomography appearance may be deceiving in instances where cysts mimic tumour necrosis seen in malignant hepatic neoplasms, and ultrasound or magnetic resonance imaging (MRI) is helpful to differentiate simple anechoic cysts from liquefied tissue.^1,3,4^

Scintigraphy with technetium-labelled sulphur colloid confirms the presence and position of either a solitary or multiple photopenic masses, as the hamartoma lacks Kupffer cells. This modality is otherwise non-specific regarding further delineation.^1^ Magnetic resonance imaging has a value in confidently establishing hepatic origin in cases of large, pedunculated, predominantly cystic lesions and to accurately delineate solid and cystic components, septations and relations to surrounding structures – especially the intrahepatic vasculature and the biliary system. Appearance may vary depending on composition. The T1 signal intensity of cystic components may differ based on the concentration of protein in the fluid but usually approximates water intensity on T2-weighted imaging. Solid components may appear hypointense to adjacent liver on both T1- and T2-weighted sequences because of fibrosis. Enhancement post-gadolinium is usually mild and confined to the septal and stromal elements.^3,4^

Differential diagnoses include benign and malignant hepatic or intra-abdominal masses, as summarised in Table 1. Occasionally, when the mass is large, the organ of origin is difficult to identify. As such, extrahepatic considerations of other cystic intra-abdominal mass lesions are often included in the differential diagnosis. These include hydatid cysts, mesenteric lymphatic malformations, gastrointestinal duplication cysts and cystic teratomas of the mesentery.^11^ Intrahepatic considerations are simple congenital hepatic cysts, which are generally rare in children and are not septated, or form part of the spectrum of polycystic kidney disease, where renal cysts are also present. Choledochal cysts are also not septated but are coupled with a degree of biliary dilation. Infectious foci, such as abscesses, have a different clinical picture with more complex internal content. Amoebic abscesses and hydatid diseases are also considered, but biochemical assessment helps in the diagnosis. Mesenchymal hamartomas with a larger degree of soft tissue may mimic cystic hepatoblastomas, the focal form of infantile hepatic haemangiomas, cavernous haemangiomas (adult-type hepatic haemangiomas), teratomas or hepatocellular carcinomas in older patients. Cavernous haemangiomas usually demonstrate a typical pattern of avid peripheral enhancement with centripetal fill-in, but differentiation could be challenging. Teratomas commonly contain fat and calcification. Calcification is also common in hepatoblastomas and infantile hepatic haemangiomas, which are more solid in nature. Alfa fetoprotein is significantly elevated with hepatoblastoma. Generally, the relatively poor enhancement of the solid components of cystic mesenchymal hamartomas helps in differentiation from these lesions. Undifferentiated embryonal sarcomas, an even rarer entity, may be indistinguishable from cystic mesenchymal hamartomas.^1,2,3,4^

**TABLE 1:** Differential diagnoses for a hepatic mass in a child.

Intra-hepatic mass		Extra-hepatic mass† (Cystic/ mixed solid-cystic lesions)
Benign	Malignant	
Hepatic haemangioma	Metastases (most commonly from neuroblastoma)	Mesenteric cyst
Congenital hepatic haemangioma	**Hepatoblastoma**	**Mesenteric lymphatic malformation (also known as lymphangioma)**
**Infantile hepatic haemangioma**	*Hepatocellular carcinoma*	Gastro-intestinal duplication cyst
*Cavernous haemangioma* (adult-type)	*Fibrolamellar carcinoma*	Mesothelial cyst
**Mesenchymal hamartoma**	**Undifferentiated embryonal sarcoma**	Choledochal cyst
Focal nodular hyperplasia	Biliary rhabdomyosarcoma	Pancreatic pseudocyst
*Hepatic adenoma*	Angiosarcoma	Cystic teratomas of the mesentery
Nodular regenerative hyperplasia	*Epithelioid Haemangioendothelioma*	Peritoneal hydatidosis
Congenital hepatic cyst		Abdominal abscess
Hydatid cyst		Cystic tumours arising from other visceral organs
Abscess (pyogenic/amoebic)		

*Source*: Chung EM, Cube R, Lewis RB, Conran RM. From the archives of the AFIP: Pediatric liver masses: radiologic-pathologic correlation part 1. Benign tumors. Radiographics. 2010 May;30(3):801–826. https://doi.org/10.1148/rg.303095173; Chung EM, Lattin GE, Cube R, Lewis RB, Marichal-Hernandez C. From the archives of the AFIP: Pediatric liver masses: radiologic -pathologic correlation part 2. Malignant tumors. Radiographics. 2011 Mar;31(2):483–507. https://doi.org/10.1148/rg.312105201; Raman SP. Mesenteric or omental mass (cystic) [Internet]. STATdx. 2016 [cited 2020 Apr 16]. Available from: https://app.statdx.com/document/mesenteric-or-omental-mass-cystic/301eb8fa-86ec-4836-9f56-89c4d67ee5d7

Note – diagnoses in bold were the primary differentials in the reported case; diagnoses in italics are generally considered in older children/adolescents.

† , Considered when the organ of origin is uncertain, particularly in pedunculated hepatic masses.

These neoplasms may undergo malignant transformation, and, as a result, complete surgical excision, with clear margins, remains the mainstay of treatment.^6^ There is a strong association with undifferentiated embryonal sarcomas, which are especially aggressive and have a poor prognosis.^12,13^ Long-term follow-up is necessary post-resection. Treatment with surgical resection is usually curative and yields admirable results. Partial lobectomy is commonly needed to remove larger tumours. The large size of these tumours makes the surgery technically challenging. If respiratory distress is the presenting complaint, percutaneous catheter drainage of large cystic elements is frequently performed as a temporising measure prior to surgical intervention.^1,3^

## Conclusions

Hepatic mesenchymal hamartoma is a rare hepatic tumour with a variable imaging appearance that has an excellent prognosis if identified and managed appropriately. In rare instances, malignant degeneration to undifferentiated embryonal sarcoma could arise whilst having a similar imaging appearance. A high index of suspicion should be asserted in all cases of asymptomatic, cystic liver masses in children and a clear understanding of the most important differential diagnoses is imperative.
